# Suitable Habitats for Endangered Frugivorous Mammals: Small-Scale Comparison, Regeneration Forest and Chimpanzee Density in Kibale National Park, Uganda

**DOI:** 10.1371/journal.pone.0102177

**Published:** 2014-07-17

**Authors:** Sarah Bortolamiol, Marianne Cohen, Kevin Potts, Flora Pennec, Protase Rwaburindore, John Kasenene, Andrew Seguya, Quentin Vignaud, Sabrina Krief

**Affiliations:** 1 UMR 7533 Laboratoire Dynamiques Sociales et Recomposition des Espaces, Paris Diderot University (Sorbonne Paris Cité), Paris, France; 2 UMR 7206 Eco-Anthropologie et Ethnobiologie (MNHN/CNRS/Paris Diderot), Paris, France; 3 Pole Image, Paris Diderot University (Sorbonne Paris Cité), Paris, France; 4 Department of Biology, Augsburg College, Minneapolis, Minnesota, United States of America; 5 Department of Botany, Makerere University, Kampala, Uganda; 6 Great Ape Conservation Project (PCGS), Sebitoli UWA Station in Kibale National Park, Fort Portal, Uganda; 7 Uganda Wildlife Authority, Kampala, Uganda; Institut Pluridisciplinaire Hubert Curien, France

## Abstract

Landscape patterns and chimpanzee (*Pan troglodytes schweinfurthii*) densities in Kibale National Park show important variation among communities that are geographically close to one another (from 1.5 to 5.1 chimpanzees/km^2^). Anthropogenic activities inside the park (past logging activities, current encroachment) and outside its limits (food and cash crops) may impact the amount and distribution of food resources for chimpanzees (frugivorous species) and their spatial distribution within the park. Spatial and temporal patterns of fruit availability were recorded over 18 months at Sebitoli (a site of intermediate chimpanzee density and higher anthropic pressure) with the aim of understanding the factors explaining chimpanzee density there, in comparison to results from two other sites, also in Kibale: Kanyawara (low chimpanzee density) and Ngogo (high density, and furthest from Sebitoli). Because of the post-logging regenerating status of the forest in Sebitoli and Kanyawara, smaller basal area (BA) of fruiting trees most widely consumed by the chimpanzees in Kanyawara and Sebitoli was expected compared to Ngogo (not logged commercially). Due to the distance between sites, spatial and temporal fruit abundance in Sebitoli was expected to be more similar to Kanyawara than to Ngogo. While species functional classes consumed by Sebitoli chimpanzees (foods eaten during periods of high or low fruit abundance) differ from the two other sites, Sebitoli is very similar to Kanyawara in terms of land-cover and consumed species. Among feeding trees, *Ficus* species are particularly important resources for chimpanzees at Sebitoli, where their basal area is higher than at Kanywara or Ngogo. *Ficus* species provided a relatively consistent supply of food for chimpanzees throughout the year, and we suggest that this could help to explain the unusually high density of chimpanzees in such a disturbed site.

## Introduction

Factors described as unfavorable to endangered species density, such as habitat fragmentation or anthropogenic activities presence, are not necessarily limiting long-term co-existence of wildlife with human activities [1], [2], [3]. Our closest relatives, the great apes, are threatened, facing decline of their suitable habitats [4]. Among them, the chimpanzee (*Pan troglodytes*) is classified on the IUCN red list as endangered and has shown a capacity to intermittently cope with human activities [5], [6] in the context of increasing proximity between wildlife and human populations [3]. Anti-poaching strategies [7], chimpanzee behavioral flexibility [8], and adaptability to environmental changes could be the main factors influencing their resilience and future capacity for long-term survival. A rough indicator of chimpanzee resilience should be their population density [9].

The availability of food resources influences the geographical distribution and population density limits of a species [10], [11]. For example, it has been shown [12] that red colobus *(Procolobus pennantii)* and redtail monkey’s group size *(Cercopithecus ascanius)* increased with food resource availability within Kibale National Park. Frugivorous/omnivorous primate (redtail monkeys, blue monkeys – *Cercopithecus mitis*, mangabeys – *Cercopithecus albigena*, l’Hoest monkeys - *Cercopithecus lhoesti*, chimpanzees, baboons - *Papio anubis*) biomass varies among sites within the park (Kanyawara, Ngogo). For example, the biomass of redtail monkeys and mangabeys was 13% greater at Kanyawara than at Ngogo, likely due to different carrying capacity of the two sites, nonequilibrium of the frugivororous community (blue monkeys were out-competed by old-growth specialists at Ngogo) and fruit availability [13], [14]. As frugivorous species, chimpanzees are strongly dependent on scarce and patchy food resources [15], [16], and thus very vulnerable to habitat disturbance [17]. During periods of fruit scarcity or in response to ecological changes, the flexible fission-fusion social structure exhibited by chimpanzees allows them to reduce party sizes (subgroup) in order to decrease potential feeding competition [18], [19], [20], [21], [22], [23], [24].

Moreover, fruit abundance is variable between seasons [25], [26] and years [27], [28] in tropical forests. Ripe fruit availability varies within primate habitats, resulting in periods of nutrient deficiency or abundance [15], [29]. Food resources can be classified by functional types, since some species provide fruit during times of high fruit abundance (HFA) while others provide fruit during times of low fruit abundance (LFA) [30]. HFA periods tend to offer a higher quantity and diversity of food, and thus frugivorous are able to choose items with preferred nutritional and chemical properties, like digestible carbohydrates and low tannins [31], [32]. Further studies have distinguished plant species fruiting during LFA periods as synchronous (sLFA) versus asynchronous (aLFA) fruit producers: the first is sufficiently abundant in the habitat during a particular fruiting season to sustain a frugivorous population [30], [33] while the second one is more constantly available but in low abundance.

Among fruit resources, figs are known to be important for chimpanzees wherever they are available [34]. For example, Fig. consumption accounts for 37% up to 90% of monthly feeding time in Kanyawara [35]. *Ficus* species are often cited as fruiting asynchronously and providing continuous and vital supply of fruits across the years for frugivorous species [27], especially during times of fruit scarcity as they serve as important fallback foods at some sites [36].

Studies performed at fine spatial scales are uniquely capable of assessing the adaptability of species to their environment [37], especially the intrinsic capability of mammals to cope with rapid environmental changes. Kibale National Park (KNP), western Uganda, is an interesting study case because it harbors a high density of Eastern chimpanzees (*Pan troglodytes schweinfurthii*, between 500–1000 individuals according to sampling methods [38], [39]) varying considerably among communities. Despite the relatively small size of the park, chimpanzee density differs greatly among different study sites [30], as observed for other primates [12], [13], [14]. The two sites experiencing the most extreme chimpanzee densities among those currently known at Kibale National Park (from 1.5 individuals/km^2^ at Kanyawara to 5.1 individuals/km^2^ at Ngogo) are separated by only 11 km.

A previous study showed the impact of spatial and temporal heterogeneity of fruit resources on chimpanzee density in the two sites [30]. Ngogo, the site with a high density of chimpanzees, experienced a high density of food plant species during a period of high productivity (HFA); plants fruiting synchronously (sLFA) during periods of low production (LFA) were also a critical component of the resource base. While there was no significant difference for the number of HFA species between sites, the number of sLFA species was higher at Ngogo than Kanyawara. This suggests that sLFA food could be an important factor promoting chimpanzee density.

In this study, we add a third site –Sebitoli-, with the goal of further understanding the link between chimpanzee density, vegetation heterogeneity and spatial and temporal food availability, providing us with a picture of the ability of chimpanzees to adapt to various ecological conditions. Sebitoli site, in the Northern part of the national park, is a very useful third case study, as it is a fragmented habitat, partly in regeneration, and surrounded by an area highly transformed by human population. Inside the forest, a tarmac road cuts through the park, while tea and eucalyptus plantations, as well as gardens, are located at the forest edge [40]. Despite these constraints, the density of chimpanzees is estimated at 4.1 individuals/km^2^ (Sebitoli Chimpanzee Project – SCP, unpublished data), which is among the highest in Kibale.

In our study, we test the following hypotheses:

First, according to the small distance between the three sites (<20 km) we expect low vegetation differences between them, and larger vegetation differences between the two more distant sites (i.e. Sebitoli and Ngogo).

Second, we assume that Sebitoli and Kanyawara are more disturbed than Ngogo because they experienced commercial logging that may have generated gaps and regenerating forests. In addition, both are located on the forest edge and thus constrained by anthropic landscape (gardens, tea and eucalyptus plantations, tea factories). If we assume that the diversity and the food availability influence the chimpanzee party size and density, food resources are expected to be smaller in Kanyawara, medium in Sebitoli and higher in Ngogo.

Third, HFA food as well as sLFA food would be higher in Ngogo (high chimpanzee density) than in Sebitoli (intermediate chimpanzee density site) and lowest in Kanyawara (low chimpanzee density site).

## Materials and Methods

### Study site

**Figure 1:**
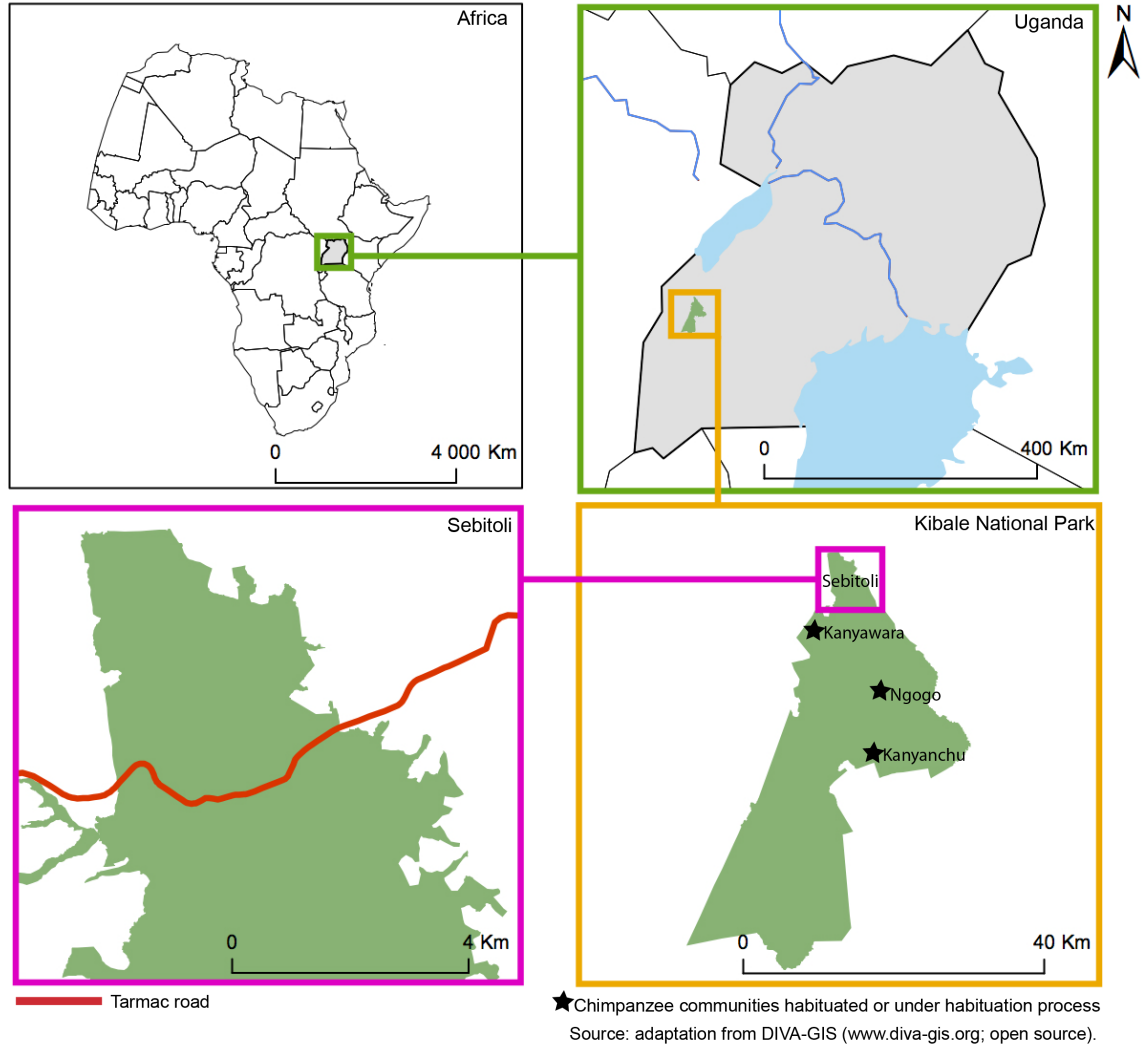
Location of Sebitoli area, Kibale National Park, Western Uganda.

Kibale National Park (795 km^2^) is located in Southwestern Uganda (0°13–0°41 N; 30°19–30°32 E; Figure 1). The park, well known for its high diversity of plants and mammals, was described as a mosaic of mature forest (58%), colonizing forest formally used for agriculture (19%), grassland (15%), woodland (6%), lakes and wetlands (2%) [41]. Local landscape is a testimony of the past exploitation of the forest (timber harvest, gardens) by the government and the local communities during the 1970’s, creating a heterogeneous landscape that greatly varies between and within sites [42].

Past forest exploitation and current anthropogenic influence reach varying intensities within the three study sites in the park (Table 1). Most of Sebitoli chimpanzee home range edges (0°36–0°40 N; 30°22–30°25 E) are in contact with anthropogenic features (32 kilometers out of 39). The management for forest exploitation defined 51 forestry compartments for the entire Kibale National Park [43]. Sebitoli (N = 11 compartments) has been commercially exploited in four compartments [43]. According to reports, logging led to about 50% of canopy opening in some areas that created forest gaps [44], [45], resulting in 35% of current Sebitoli area being harvested [46]. No detail about intensity of logging in Sebitoli has been published. The same proportion of surface area has been harvested in Kanyawara [46] (0°33–0°36 N; 30°20–30°23 E, N = 7 compartments) but contrary to Sebitoli, information of logging activities within the distinct units has been well documented [44], [45], [47]. From the literature we know that various compartments were lightly logged (K14), heavily logged (K15), and unlogged (K30) compartments [44], [45], [47], [48]. In comparison, Ngogo (0°28–0°30 N; 30°22–30°26 E, N = 3 compartments) seems more homogenous than the two other study sites because it there has been no logging, but historically there have been human settlements, resulting in large grasslands in today’s landscape [49]. Therefore, the combination of the features of the three sites gives a precise indication when studying variables influencing chimpanzee densities.

**Table 1:** Past and present anthropogenic influence [30], [38], [50], [51].

	Sebitoli	Kanyawara				Ngogo
		K14 (Lightlylogged)	K 15 (Heavilylogged)	K 30(Control)	Total	
	Past					
Timber harvest	x	x	x		x	
Human settlements	x				x	x
Slash-and-burn cultivations	x	x	x	x	x	x
	Present					
Number of tea factories atproximity (>500 m fromedge)	3	0	0	0	0	0
Tarmac road	x					
Home range in contact with gardens	81.6%	7.5%	0%	1.2%	47.6%	0%

Within Kibale National Park, there is a north-south gradient in elevation causing an increase in temperature and a decrease in rainfall from North to South [45]. The Kanyawara chimpanzee community is located almost in the middle of the two other sites: Sebitoli and Ngogo (maximum distance between Sebitoli and Ngogo community centroids: 17 km).

### Ethics statement

The studied chimpanzees were observed without any invasive methods or contacts with researchers. Methods used to collect data are in compliance with Uganda Wildlife Authority guidelines and keep to the legal requirements of Uganda. All necessary permits were obtained for this study. The research proposal and the field study is conducted under a “Memorandum of Understanding for research and conservation of chimpanzees in Kibale National Park” between National Museum of Natural History (legal department, SJ445-12), Uganda Wildlife Authority and Makerere University signed July 16^th^, 2012 for 10 years.

### Sebitoli chimpanzee community composition

Chimpanzee habituation in Sebitoli began in 2008 under the Sebitoli Chimpanzee Project.

The field teams of the SCP are composed of two to eight people (Field assistants – FA, researchers and students) and organized in one to four groups. A grid of 80 km of trails is used daily by the research teams. FAs were trained by S Krief to collect data related to chimpanzees and vegetation. Data related to chimpanzees are collected every day, starting at 6 a.m., for 12 hours per day. In this study, four years of Sebitoli data collected by SCP (from February 2^nd^, 2009 to January 29^th^, 2013) are analyzed.

Evidence of chimpanzee presence (feces, direct observations, nests, footprints, vocalizations) was used to locate and characterize chimpanzee groups. During the habituation period, chimpanzee location and behaviors were recorded *ad libitum* and contacts, as well as signs of presence, were geo-refererenced (GPS Garmin Oregon™ 300, 400, 450). SCP team recorded the following data systematically: distance between chimpanzee(s) and observers, orientation, tree height, number of individuals, identity (if possible), activities, health status and behaviors, response to observers, level of habituation, food species (item consumed, and its maturity). All GPS points were geo-referenced in the same geodesic system (WGS 84) and cartographic projection (UTM 36 N).

In January 2013, Sebitoli chimpanzee community was estimated to be composed of one hundred individuals, of which 79 were identified, including 28 adult females and 18 adult males (categories according to [52]).

Minimum Convex Polygon method was used to define the Sebitoli chimpanzee home range (MCP-2051 points collected by SCP team during the study period; ET Geowizard software, ArcGIS 9.3). To quantify the Kanyawara chimpanzee home range, MCP method was also used with 2 546 points collected by S Krief and two field assistants during previous research (12 years of data). Finally, MCP generated by SJ Amsler [53] was used to define the Ngogo community (3 901 points, 2003 to 2005 period).

### Sebitoli chimpanzee diet

Fresh food-remains and seeds found in fresh feces (less than six hours old) during chimpanzee monitoring, were considered as species consumed by chimpanzees. Due to the habituation process and the *ad libitum* data collection, a feeding bout was considered to begin when at least one chimpanzee of a party was consuming an item of food and to end when all chimpanzees of the party had stopped eating. In addition, party membership was continuously assessed. To estimate the consumption frequency of the different items, the length of feeding bouts related to one item (duration) was multiplied by the number of chimpanzees in the party consuming that food item.

The list of species consumed by chimpanzees was obtained at Sebitoli after 4 years *ad libitum* observations and compared to Kanyawara and Ngogo’s data gathered from long-term, published and observed data [30]. We first considered a set of the 18 fruiting species most commonly consumed by the chimpanzee community in each site (some species are common and others are different between sites). We further focused on a set of the top (most consumed) seven species out of the 18, corresponding to 90%, 60–80% and 75% feeding-time at Sebitoli, Kanyawara and Ngogo respectively.

### Land-cover composition

Land-cover, not studied in the previous survey [30], was established with identical remote sensing methods for the three sites in a comparative perspective. We used satellite images to evaluate vegetation type diversity, habitat types and their proportions in Sebitoli, Kanyawara and Ngogo. Since land-cover diversity throughout the forest could affect spectral classification inside the forest, as well as its quality, remote sensing analysis was restricted to the forested areas composing each chimpanzee home range. A mask of each site using MCP of respective community home ranges was built. Envi 4.8 was used in remote sensing treatments based on Landsat 7 image (ETM+, orthorectified, 14/03/2001, 30 m) to spectrally determine habitat composition. The following protocol was applied:

1. Principal Component Analysis (PCA) on spectral bands.
2. Unsupervised classification. Five habitat classes were discriminated using K-means method (10 iterations) based on neo-canals obtained from PCA. This allowed us to examine a precise and common classification of habitat types for each study site according to spectral radiometric curves analysis and our knowledge of Sebitoli and Kibale in general. From habitat types defined in literature [41], we can precise the previous classifications by analyzing chlorophyll activity intensity (peak on band 4) and by visual interpretation of woody/non-woody species density gradient to discriminate habitat types that were categorized as follows: Terrestrial Herbaceous Vegetation (THV - herbs and small woody shrubs, abundant and evenly distributed resources belonging to the Zingiberaceae, Marantaceae, Gramineae and Acanthaceae families [16]), degraded forest, regenerating forest, mature forest and grassland areas (mostly represented in Ngogo, very open areas with low chlorophyll activity).

### Spatial variation in food availability

Botanical composition was surveyed inside the forest within 63 plots located in Sebitoli chimpanzee home range according to land-cover classes (using an adapted and comparable protocol from [30]). Plots were placed randomly using a stratified sampling method [54] where the number of plots is proportional to surface areas of each land-cover class previously defined with Landsat image.

Nested strip widths of 50 and 20 m were used to enumerate and measure all stems of different size classes and growth forms (even the ones not included in chimpanzee diet).

All trees and free-standing stems with a diameter at breast height (d.b.h., measure of the diameter of a trunk at 1.30 m) greater than 30 cm (large-size stems) and all strangler figs with a d.b.h. greater than 10 cm were identified and measured in the large size plot (50×50 meters plot).
All trees and free-standing stems with a d.b.h. between 10 cm and 29 cm (medium-size stems) were identified and measured in the medium-size plot (20×50 meters).

Within the 63 plots, large-size stems (≥30 cm) were recorded in 15.75 ha, and intermediate-size stem (10 to 29 cm) in 6.3 ha. The total proportions of vegetation classes is very similar to previous study [1] but the surface area per d.b.h. class differ mostly for d.b.h. between 30 and 80 cm.

S Bortolamiol and two SCP FA conducted plot censuses. They prepared herbaria in triplicate. One set of herbaria was studied at Makerere University Botany Herbarium Department by P Rwaburindore and J Kasenene.

### Temporal variation in food availability

Temporal variations in food availability in the Sebitoli chimpanzee home range were estimated through data obtained from phenological surveys conducted each month by FA between February 2012 and July 2013 (18 months). During the study period, data were collected along 10 trails of 500 meters long each (i.e 5 km in total) dispatched in the Sebitoli chimpanzee home range. 528 individuals of 47 species were monitored at Sebitoli. FA were trained to note the maturity of items (leaves, flowers and fruits) and to give abundance scores (ranging from 0 to 4, 0 representing no fruit and 4 representing a maximum fruit concentration). To analyze resource availability in the forest, food availability of individuals capable of fruiting, bearing unripe and ripe fruits was calculated. Only the feeding species with at least three individuals monitored along the trails that were also represented in vegetation plots were considered. A d.b.h. measurement was taken for all feeding trees on the trail.

### Quantifying fruit abundance

In order to compare the density and heterogeneity of fruit-bearing vegetation between sites, the spatial fruit abundance at Sebitoli was determined using a methodology inspired by the one applied to Kanyawara and Ngogo [30]. To sum up the following steps, a schematic representation of basal area and Food Availability Index (FAI) was designed (Figure S1).

#### (1) Fruit abundance in space

We calculated basal area per hectare of the 18 most consumed species as well as HFA, sLFA or aLFA species in function of their d.b.h. size (G’: ≥30 cm d.b.h.; G”: 10 cm ≤ d.b.h. <30 cm on Figure S1).

#### (2) Fruit abundance in time

Using phenology records, the temporal fluctuation in monthly fruit abundance at Sebitoli was assessed using a ‘percent basal area fruiting/ha’ method [30], [55], and the following formula:

FAI = ∑(G(plots) *x* number of stems bearing fruits per species per month (phenology)).

This method enables a comparison of data across sites by limiting differences in surface area covered by phenology transects and the composition of individual trees monitored.

A monthly score was obtained and classified using a percentile ranking method. Months below the 25^th^ percentile were classified as Low Fruit Abundance (LFA), months higher than the 75^th^ percentile were classified High Fruit Abundance (HFA) and months with an intermediate score were classified Intermediate Fruit Abundance (IFA).

Next, the availability of fruiting species monitored in phenology was classified HFA when more than 30% of individuals were fruiting in HFA months, and less than 20% in LFA months. Species with less than 30% individuals fruiting in HFA months, and more than 20% in LFA months were classified as LFA [1]. For LFA species, a dispersion index - Green Index [56]- was then calculated (PaSsage software) to divide LFA species in 2 categories: synchronous (sLFA) and asynchronous (aLFA).

As our study aims to compare results (see [30]), the same calculation methods were used for species functional classes. However, species fruiting mostly during IFA months did not fit criteria that were just defined in this study. Therefore, IFA species were classfied (N = 6, *Ficus exasperata, Ficus sur, Ficus sansibarica, Prunus africana, Eudenia eminens, Ficus mucuso*) as having LFA or HFA tendencies if any two of the following three criteria applied to the species: percentage of HFA/LFA higher than percentage of LFA/HFA (criteria 1), HFA or LFA percentage deviation closer to the mean (criteria 2) and number HFA/LFA months with no fruit (criteria 3).

Only *Eudenia eminens* could not be classified with this method and was defined as IFA species. Contrary to Ngogo, *Ficus mucuso* are very rare in Sebitoli chimpanzee home range (N = 5 known by SCP in the entire home range) and no individual was recorded in our vegetation plots. However, the fruit of this species is one of the ten most consumed items in the Sebitoli chimpanzee diet, and is likely a very important resource for this community. Therefore, to include this species in the analysis, basal area of *Ficus mucuso* monitored in phenology was used.

Basal area of the 18 species (including top seven fruit-providing species) being most consumed by chimpanzees at each site was compared using Mann-Whitney test followed by Monte-Carlo simulations (10 000 iterations) using XLStats software and Sebitoli, Kanyawara and Ngogo data [30]. Basal areas of HFA, sLFA and aLFA species were also compared. As a unit of the analysis, basal area of stems in each botanical plot was used.

### Relation between feeding patch and party size

A feeding patch was defined as an aggregation of food items that allowed uninterrupted feeding for an individual or a party [57]. For most frugivorous species, including chimpanzees, a feeding patch can often be operationally defined as a single tree, where the size of the tree (d.b.h.) represents the size of the feeding patch. The relation between feeding patch size and number of chimpanzees in the party at Sebitoli was tested using a linear regression (XLStats).

### Species diversity

Using vegetation plots and Landsat classification, species diversity per hectare of trees was computed by size class (10≤ d.b.h. <30 cm, 30≤ d.b.h. <80 cm, d.b.h. ≥80 cm), for all tree species and for the top 18 species consumed, separately for each of the five land-cover classes. Shannon (H) index was used to measure diversity (Past software, version 2.6). Mean values of H were used as a general indicator to compare sites because there were not enough plots placed in some land-cover classes (especially grasslands at both sites, and THV at Ngogo) to meet the assumptions of standard statistical tests.

## Results

### Land-cover properties

**Figure 2:**
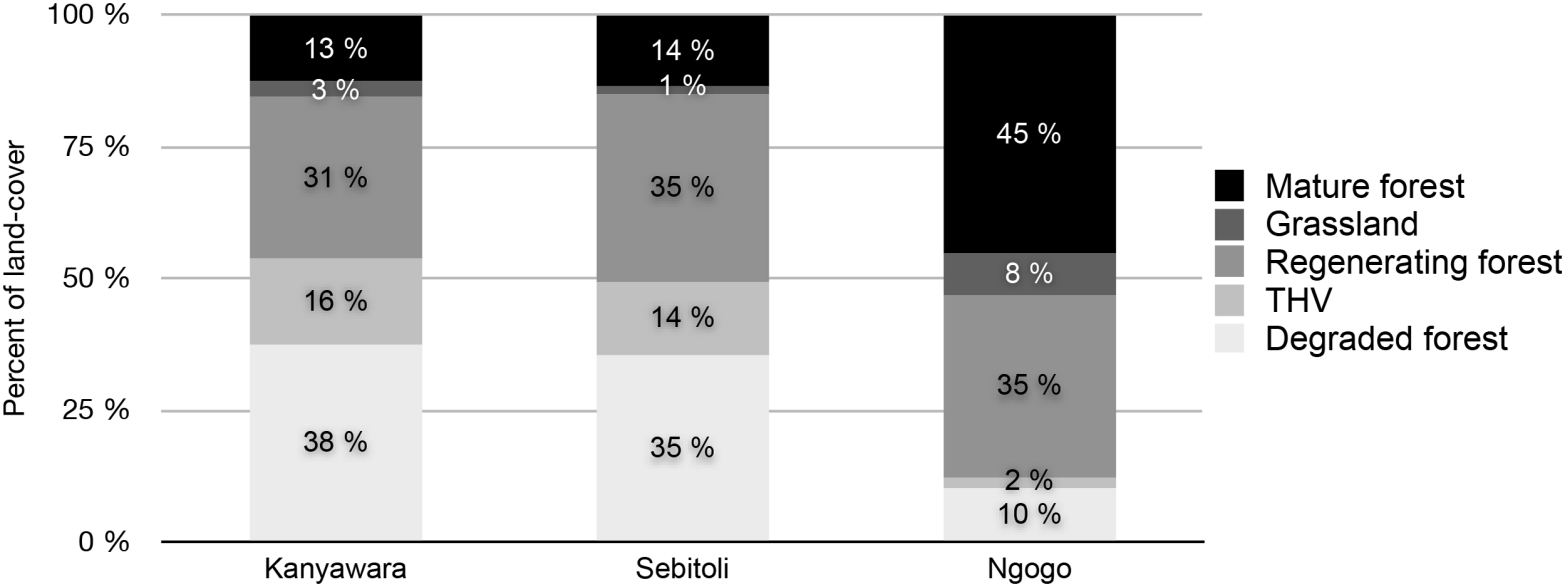
Sebitoli land-cover in comparison with Ngogo and Kanyawara.

Sebitoli and Kanyawara land-cover compositions are quite similar (mainly degraded vegetation and regenerating forest), and differ from that of Ngogo (mainly mature and regenerating forest) (Figure 2).

### Tree diversity at Sebitoli

**Figure 3:**
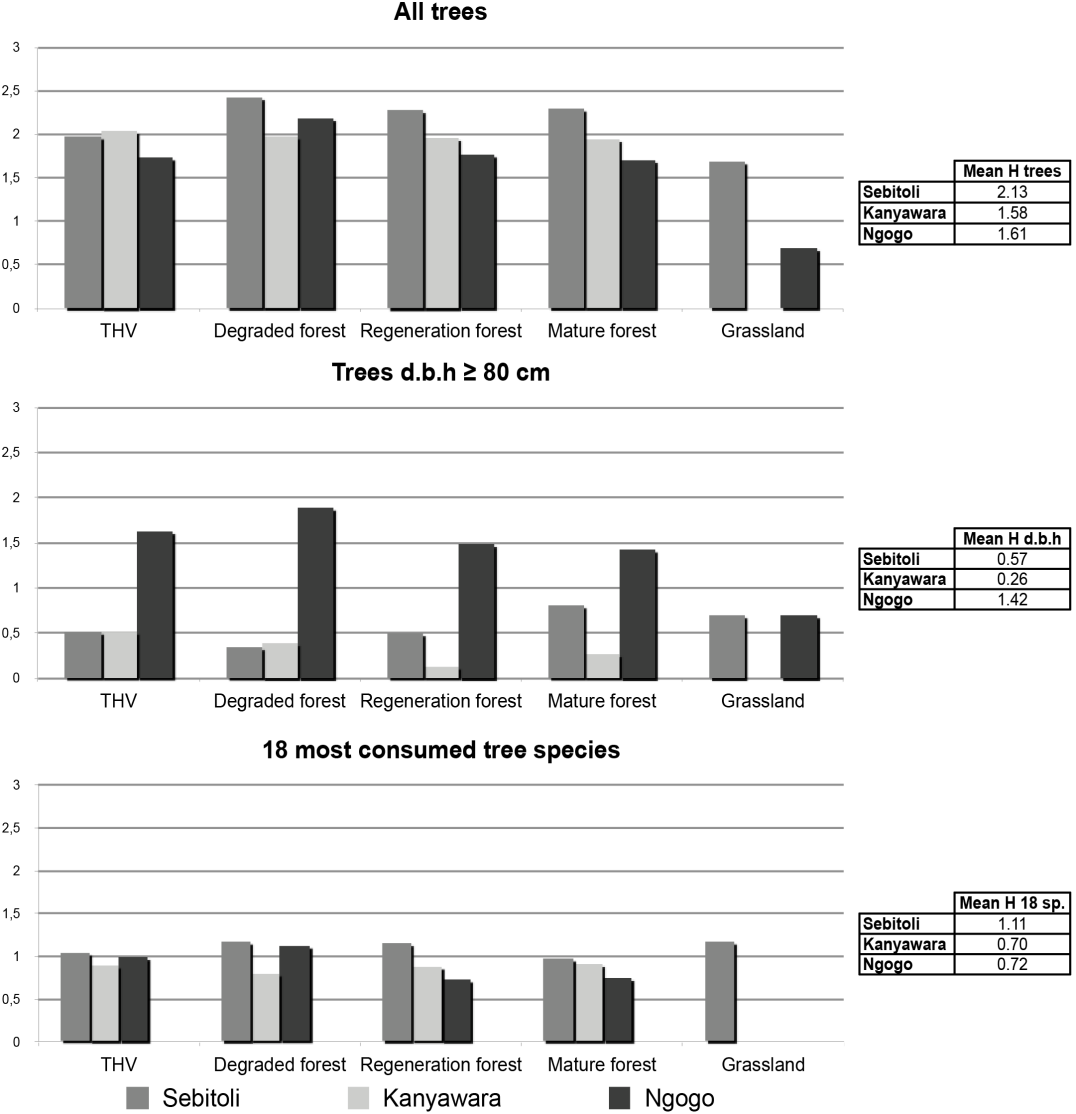
Diversity index in function of habitat types and vegetation characteristics.

We computed and compared vegetation diversity in each land-cover class previously defined at Sebitoli, Kanyawara and Ngogo (Figure 3).

There are clear differences in diversity among tree class sizes. Trees with a d.b.h. greater than or equal to 80 cm were far more diverse at Ngogo in most land-cover class (except for grassland) compared to Sebitoli and Kanyawara. For all trees, and among the 18 most consumed tree species recorded in vegetation plots, species diversity (H) is generally higher at Sebitoli than Kanyawara and Ngogo (Figure 3). Kanyawara is far less diverse for regeneration and mature forest, and is generally less diverse across habitat types compared to Sebitoli.

### Sebitoli chimpanzee diet

During the study period, hours spent per year (1490–3344) and number of teams in the forest increased, showing an intensive effort from FA and researchers to locate chimpanzees during the habituation process. The efficiency of chimpanzee habituation is indicated by the increase in visual contact hours per year between observers and chimpanzees (132–1370 hours) and in mean contact time with chimpanzees per year (36–126 minutes), respectively, by multiples of 10 and 4 between February 2009 and January 2013.

Through the study period, observations of feeding bouts (129–561) and time feeding (55–514 hours) increased, which also suggests progress in the habituation process. Mean party size during feeding activities was variable, ranging from 3.56 to 5.35 individuals (range: 1–21).

Using four years of *ad libitum* observations recorded during the habituation process, 89 food items were counted including 17 THV species (23 items; N_species_ = 9 for piths, 1 stem, 2 flowers, 6 leaves, 5 fruits) and 52 tree species (66 items; N_species_ = 4 for piths, 4 bark, 1 dead wood, 1 wax, 5 flowers, 11 leaves, 40 fruits) (Tables S1, S2) consumed by Sebitoli chimpanzees.

### Link between party size and feeding patch size

Only a very small percentage of variation in party size at Sebitoli was explained by feeding patch size (linear regression R^2^ = 0.059, P-value <0.0001). Also, feeding patch size is smaller at Sebitoli (55.31 cm d.b.h.) than Kanyawara (66.87 cm) and Ngogo (63.38 cm).

### Intersites comparison of food resources availability

In order to compare spatial and temporal availability of the 18 fruiting trees most consumed by chimpanzees, we compared the sum of their basal area and functional types (HFA, aLFA, sLFA) in the three communities.

**Figure 4:**
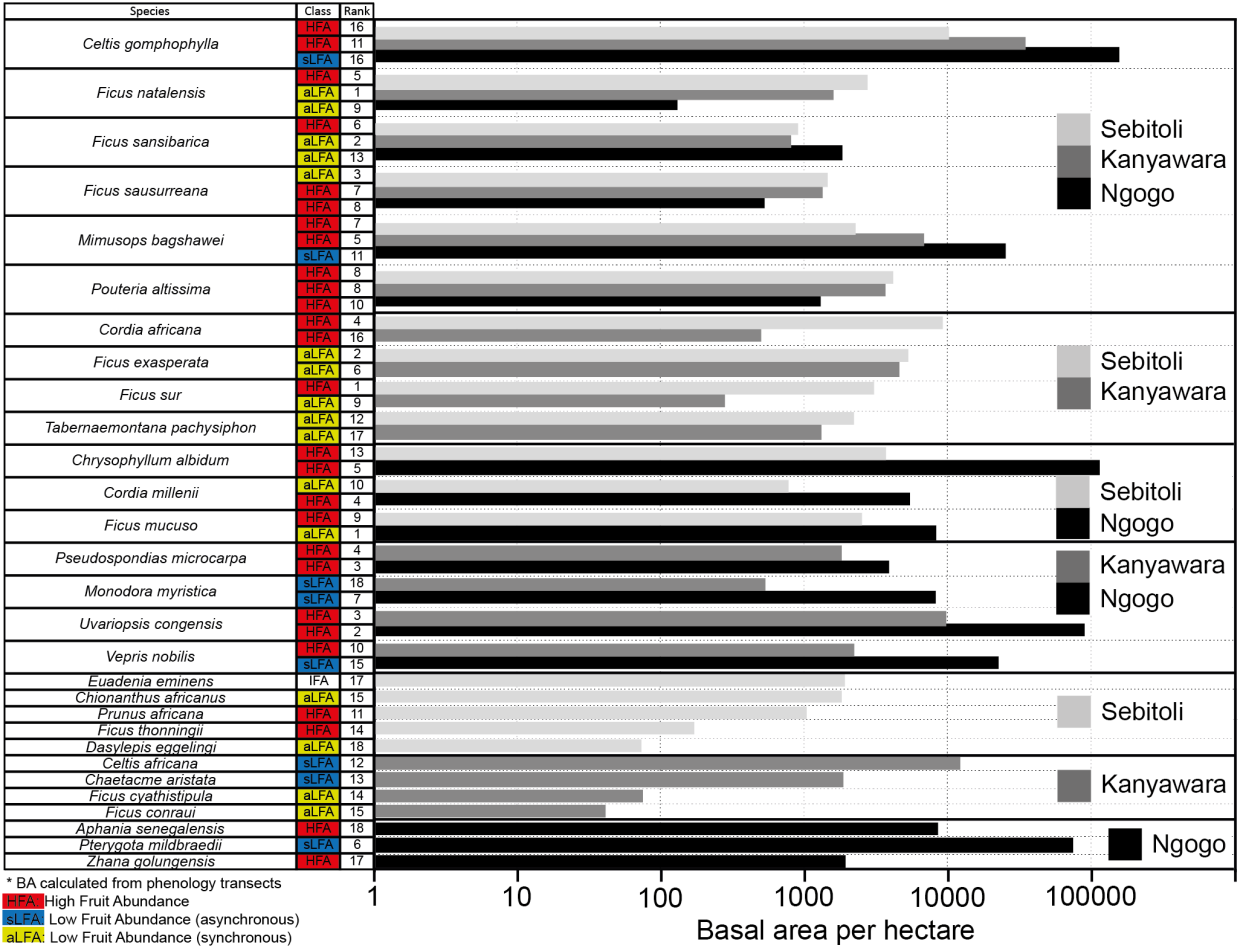
Sum of 18 most consumed species basal area, fruit availability and consumption rank at Sebitoli, Kanyawara and Ngogo (data for Kanyawara and Ngogo from [30]). *Morus lactea* (sLFA) and *Treculia africana* (HFA) at Ngogo were absent from plots but present in Ngogo chimpanzee diet.

The sum of the basal area of the 18 most consumed fruiting trees is respectively 1.5 and 9.5 times lower at Sebitoli (54 683 cm^2^/ha) than at Kanyawara (83 553.9 cm^2^/ha) and Ngogo (519 175.9 cm^2^/ha) (Figure 4). Among the top 18 species consumed at Sebitoli, 10 species are shared with Kanyawara (six of them shared the same temporal availability with Sebitoli), nine species are shared with Ngogo (two of them shared the same temporal availability with Sebitoli) and there are six species found at all three sites. Seven species at Sebitoli (17 627.3 cm^2^/ha) and Kanyawara (8 666.8 cm^2^/ha) belong to the *Ficus* genus whereas there are only four *Ficus* species among the top 18 food species at Ngogo (10 708.6 cm^2^/ha).

Among the 18 most consumed species, the proportion of HFA species is greater at Sebitoli (N = 11) than at Kanyawara (N = 8) and Ngogo (N = 9). Also, there are no sLFA species at Sebitoli while there are three at Kanyawara and six at Ngogo. One species was classified as IFA at Sebitoli and showed no particular pattern of fruiting. aLFA species were more various at Kanaywara (N = 7) than Sebitoli (N = 6) or Ngogo (N = 3). Finally, at Sebitoli, five *Ficus* species out of seven are classified HFA while there is one out of four at Ngogo and one out of seven at Kanyawara. Other *Ficus* species were aLFA at the three sites.

According to the Mann-Whitney tests (Table 2), Ngogo shows generally higher mean and median basal area per hectare than Sebitoli and Kanyawara for the top 18, the top seven and the HFA species. However, Sebitoli shows significantly higher mean basal areas for overall (11 529.6 cm^2^/ha), intermediate (5 378.8 cm^2^/ha) and small size stems (3 152.4 cm^2^/ha) of aLFA compared to Kanyawara (6 798.0 cm^2^/ha, 2 453.6 cm^2^/ha, 1 441.8 cm^2^/ha respectively) and Ngogo (4 439.1 cm^2^/ha, 0 cm^2^/ha, 0 cm^2^/ha respectively).

**Table 2:** The Mann-Whitney test results on basal area of 18 species being most consumed by chimpanzees (data for Kanyawara and Ngogo from [30]), top seven food species and the different categories of food resources.

		Ngogo			Kanyawara			Sebitoli			Mann-Whitney tests						
	Category	MeanBA/ha(cm^2^ ha−1)	MedianeBA/ha(cm^2^ ha−1)	SE	MeanBA/ha(cm^2^ ha−1)	MedianeBA/ha(cm^2^ ha−1)	SE	MeanBA/ha(cm^2^ ha−1)	MedianeBA/ha(cm^2^ ha−1)	SE	MWUKN	P-valueKN	MWUSK	P-valueS/K	MWUSN	P-valueS/N	DirectionS/N/K
**Top 18**	Overall	157875.2	132622.4	16511.7	83752.5	74038.4	8409.3	54683.0	45238.9	5698.3	834.0	0.0000	1162.0	0.0062	661.5	**<0.0001**	N>K>S
	Large trees	39919.0	22698.0	8461.0	14807.7	0.0	3989.7	21451.2	0.0	1082.8	1063.0	0.0450	1725.5	0.5848	1376.5	0.1172	N>S≈K
	Intermediate trees	57128.6	43568.5	6156.8	43181.9	32114.3	5037.4	24074.6	19267.4	794.1	1085.0	0.1150	1109.0	0.0030	868.0	**<0.0001**	N≈K>S
	Small trees	60827.6	52400.2	6422.4	25763.0	20998.5	4140.0	9157.2	7647.0	113.5	681.5	**<0.0001**	1160.0	0.0064	345.5	**<0.0001**	N>K>S
**Top seven**	Overall	104763.9	88911.3	13462.7	25567.5	1836.2	6390.6	26360.8	7450.9	4766.8	626.0	**<0.0001**	1838.0	0.2396	815.0	**<0.0001**	N>S≈K
	Large trees	26213.3	0.0	5769.5	9039.6	0.0	2561.0	14190.6	0.0	1017.3	910.0	0.0010	1628.0	0.9490	1301.0	0.0298	N>S≈K
	Intermediate trees	32620.2	20434.2	4655.9	5476.5	0.0	1905.9	11168.2	4071.5	623.0	278.0	**<0.0001**	2117.0	0.0024	1007.0	0.0004	N>S>K
	Small trees	45930.5	27354.3	6733.2	11051.3	0.0	3568.9	1002.0	0.0	35.3	329.5	**<0.0001**	1392.0	0.0614	527.0	**<0.0001**	N>K≈S
**HFA**	Overall	72055.3	65737.5	9489.5	62455.8	58738.3	7412.0	37485.0	29303.2	5306.4	1285.5	0.6600	1116.5	0.0026	1220.5	0.0220	N≈K>S
	Large trees	5677.9	0.0	2072.4	10214.1	0.0	3913.7	17410.5	0.0	1065.0	1394.0	0.6290	1884.5	0.0726	1968.0	0.0190	S≈K≈N
	Intermediate trees	20447.0	8073.9	3751.2	33014.3	21384.9	4305.7	15530.0	7543.0	632.4	1756.0	0.0070	997.0	0.0002	1589.0	0.7716	K>N≈S
	Small trees	45930.5	27354.3	6733.2	19227.4	5291.2	4031.5	4544.4	1539.4	86.9	916.5	0.0030	1212.0	0.0128	736.0	**<0.0001**	N>K>S
**sLFA**	Overall	81380.7	73868.2	9644.8	14498.7	7228.3	2433.6	0.0	0.0	0.0	340.0	**<0.0001**	-	-	-	-	Only in N
	Large trees	29802.0	0.0	7894.4	1691.0	0.0	959.4	0.0	0.0	0.0	879.5	**<0.0001**	-	-	-	-	Only in N
	Intermediate trees	36681.6	33560.2	4367.1	7714.0	0.0	1553.4	0.0	0.0	0.0	609.5	**<0.0001**	-	-	-	-	Only in N
	Small trees	14897.1	6372.4	2745.9	5093.8	0.0	1283.5	0.0	0.0	0.0	936.0	0.0034	-	-	-	-	N>K
**aLFA**	Overall	4439.1	0.0	1924.2	6798.0	0.0	1811.8	11529.6	6605.2	1932.3	1646.0	0.0224	2323.0	0.0006	2591.0	**<0.0001**	S>K>N
	Large trees	4439.1	0.0	1924.2	2902.5	0.0	964.3	2998.5	0.0	325.5	1402.0	0.6632	1423.5	0.0518	1488.0	0.1876	N≈S≈K
	Intermediate trees	0.0	0.0	0.0	2453.6	0.0	1065.6	5378.8	0.0	265.5	1508.0	0.0254	2199.0	0.0004	2444.0	**<0.0001**	S>K
	Small trees	0.0	0.0	0.0	1441.8	0.0	574.1	3152.4	1606.1	52.3	1560.0	0.0060	2300.0	**<0.0001**	2626.0	**<0.0001**	S>K
**IFA**	Overall	-	-	-	-	-	-	1898.4	0.0	482.9	-	-	-	-	-	-	Only in S
	Large trees	-	-	-	-	-	-	319.1	0.0	81.1	-	-	-	-	-	-	Only in S
	Intermediate trees	-	-	-	-	-	-	344.7	0.0	45.6	-	-	-	-	-	-	Only in S
	Small trees	-	-	-	-	-	-	1234.6	0.0	32.2	-	-	-	-	-	-	Only in S

Direction compares BA sum and indicates p-value difference significativity (p-value ≤0.05; < or >) or non-significativity (≈) (SE: Standard Error).

Between Sebitoli and Kanyawara, there was only one aLFA size category showing a significant difference between sites (small stems, P-value <0.0001). The difference was more pronounced between Kanyawara and Ngogo, for which seven size categories showed significant differences (each P-value <0.0001). This trend was even more pronounced between Sebitoli and Ngogo as nine categories were signicantly different (P-value <0.0001). Finally, standard errors (SE) were consistently lower at Sebitoli than Kanyawara and Ngogo.

## Discussion

Despite their proximity and similar sizes, the three chimpanzee study sites in Kibale National Park that we studied here differ considerably in terms of food-resource species availability (1), temporal fluctuation (2) and tree maturity (3).

Sebitoli and Kanyawara have similar land-cover characteristics, but Sebitoli is essentially a cul-de-sac surrounded by tea plantations and crossed by a road. Basal area of the 18 most commonly consumed species by chimpanzees were 9.5 times higher at Ngogo than at Sebitoli. Our results confirm that the sites furthest from one another are also the most distinct in terms of landscape-scale vegetation characteristics (i.e. Sebitoli and Ngogo, hypothesis 1 - confirmed).

Sebitoli harbors a higher density of chimpanzees than Kanyawara. Nevertheless, during our study period, food patch sizes and party sizes were small at Sebitoli compared to Kanyawara and Ngogo [58]. However, our results suggest that it is possible that chimpanzee density is more closely related to diet composition than to food abundance, per se, with *Ficus* and tree species diversity playing key functions. Among the most consumed species at Sebitoli, five of the top seven species and eight of the top 18 species are *Ficus* species. Those *Ficus,* being important to sustain chimpanzee diet year-round [59], may have developed after forest exploitation in Sebitoli, which, as Kanyawara, was affected by such actions. From Sebitoli vegetation plots, 8 species of *Ficus* were censussed (N = 69 individuals) and most individuals were hosted in degraded (N = 24) and regeneration (N = 23) forests. Also, *Ficus* species do not behave the same way: some species such as *Ficus sur* may have multiplied after forest exploitation because their d.b.h. is smaller (min: 35, max: 104, mean 62.3, SD: 22.47) compared to species such as *Ficus saussureana* (min: 95, max: 141, mean: 118, SD: 32.53) that are generally larger and may have prospered after logging. They represent the main food resource for Sebitoli chimpanzees because they are abundant (HFA) and available all year long (IFA, aLFA). Also, tree species diversity is generally higher at Sebitoli than at Kanyawara and Ngogo for all trees in each habitat types as well as for consumed species (Hypothesis 2 – partly confirmed).

Finally, while sLFA species were described as possibly promoting high chimpanzee density at Ngogo compared to Kanyawara, they are totally absent from Sebitoli (Hypothesis 3 – partly confirmed).

### Landscape differences and chimpanzee diet

We found larger differences in floristic composition between more distant sites, Sebitoli has more species in common with Kanyawara (closer study site) than Ngogo. While Sebitoli and Kanyawara are similar in terms of land-cover composition, Fig. trees are more dense and diverse at Sebitoli. Also, *Ficus* basal area (among the 18 most heavily consumed items) is higher at Sebitoli than at Kanywara and Ngogo. Sebitoli and Kanyawara border the forest edge (Figure 1) while Ngogo is located in the middle of the forest. Gaps and edges caused by logging activities can favor sunlight, fruit production of the tree crowns and increase average of leaf quality [60] as well as growth of terrestrial herbaceous vegetation and Fig. trees. Previous studies showed that plant species richness was higher in area previously deforested than in non-exploited areas, and these areas were used by primates [41], [61], [62].

Trees basal area in Kanyawara increased in all compartments between 1989 and 2006 [61] but previous studies showed that growth rates were variable in function of past logging activities: trees in the most heavily logged area had the slowest growth rate of any of the areas and trees in moderately and lightly logged areas had a slightly faster growth rate than either of the unlogged areas [48]. However, in a study related to red colobus monkeys, analysis on each compartment of Kanyawara indicated that the cumulative d.b.h. of food trees increased in the heavily logged (P = 0.003) and lightly logged area (P = 0.008) but not in the unlogged forest (P = 0.191) [61]. Therefore, logging activities impact forest structure and primate feeding trees to different extents.

Some primate species, such as redtail monkeys and red colobus live on forest edges/patches which is possibly due to dietary preferences for second growth forests and their higher abundance in those areas maybe due to colonizing plants [62]. Mean group densities for frugivorous such as redtail monkeys and gray-cheeked mangabeys were higher in lightly logged than unlogged areas in Kanyawara [63]. Kanyawara chimpanzees use forest compartments that were logged in different intensities (K14, K15, K30) and female individuals mostly range in lightly logged (K14) and unlogged (K30) areas [64]. At Budongo, logged areas and forest edges provided 76% of the chimpanzee food while they represent only 60% of chimpanzee home range [65].

The basal area per hectare of *Ficus* species is higher at Sebitoli (15196.31 cm^2^) than at Kanyawara (8187.63 cm^2^) and Ngogo (5379.87 cm^2^) and *Ficus* species represent a higher percentage among all stems at Sebitoli (7.46%) than at Kanyawara (2.81%) and Ngogo (1.56%). Within Kanyawara, *Ficus* density within all stems increased with logging intensity (K30: 1.43%; K14: 2.43%; K15: 2.69%). Their current basal areas in compartments that were logged in the 1970s is more important in lightly logged areas (K14: 16051.19 cm^2^) than in unlogged (K30: 5104.61 cm^2^) or heavily logged (K15: 3407.09 cm^2^). Low intensity selective logging could be compatible with the conservation of primates [61]. It is possible that commercial timber harvesting did not have a major long term influence on the critical resource base of chimpanzees at Kanyawara and Sebitoli and that the differences of feeding resources between sites result from natural heterogeneity and logging activities [45], [66]. It was suggested that light penetration to the forest floor was higher at Kanyawara than Ngogo, favouring the establishment of light demanding species [48]. The fact that *Ficus* basal area and stem proportion are more important at Sebitoli than the two other sites within all trees (and then fruit production) is to be considered as a factor of chimpanzee density. Figs represent relatively high quality food necessary to sustain chimpanzees during times of overall fruit scarcity and this could account for the difference in chimpanzee density between Sebitoli and Kanyawara. There are differences between the pulp and the seed component of Fig. trees (higher caloric density) and Kanyawara figs were described as “energy-rich food with adequate protein” [34]. With regard to the nutritional value of figs, researchers have said that “they should be considered as potatoes for humans, a food that will sustain life at maintenance” [67] meaning that figs are a staple food item to chimpanzees.

### Perspectives on chimpanzee adaptability to anthropogenic changes

Sebitoli, the site of intermediate chimpanzee density, had a higher density (at least for the seven species most consumed) and diversity of food resources than Kanyawara (low chimpanzee density). As at Kanyawara, Sebitoli was logged and the lower standard error values in basal areas among stems compared to Kanyawara and Ngogo suggest that most of the forest at Sebitoli is in the same successional stage (smaller d.b.h. compared to Ngogo). Therefore, it is possible that since food patch size (d.b.h.) is smaller at Sebitoli compared to Kanyawara and Ngogo (largely attributable to past forest exploitation), fruiting species can sustain fewer chimpanzees on a tree. Using a linear regression, we found no dependence between the feeding party size (FPS) and food patch size at Sebitoli during the study period. While food patch size explained a large part of the variance in FPS at Ngogo (80%), it explained far less of this variance at Kanyawara (22.7%) [58] and it only explained 5% of the variance in FSP at Sebitoli. Indeed, if fruit resources are consistently available through the year and high-quality patches sparsely distributed, no relationship between FPS and patch size should exist [15].

In the three sites, chimpanzees have long coped with various forms of anthropogenic change (e.g. agriculture, fire, logging). Increasing fragmentation [2] and proximity between natural and anthropogenic landscapes have resulted in close co-existence between wild animals and human populations. Some individuals from different chimpanzee communities are known to cross roads passing through their home range (Bossou, Guinea: [6]; Sebitoli: SCP unpublished data), to raid crops in neighborhood gardens [5; SCP unpublished data], and even deactivate snares set by poachers [68].

The fact that adult chimpanzees continue to observe and learn the use of unfamiliar feeding items from conspecifics with potentially better fitness (males and females between 25 and 40 years old) [69] suggest that chimpanzee adaptation to novel environments is a potentially long-term process based on social transmission. Investigating behavioral and genetic characteristics of migrating females would enable us to monitor the ability of chimpanzees to adapt to environmental changes and their capacity for resiliency, especially under such intense anthropogenic constraints as they currently face (roads, human settlements, threats of snare injuries, etc.).

### Inter-sites variations in temporal food availability for chimpanzees

Comparisons between sites within the same forest block are not as common as comparisons between primate populations inhabiting different forests [13], [42]. Based on our results, we can conclude that both the density and the temporal availability of food resources for chimpanzees may impact on chimpanzee density in KNP. HFA and sLFA species apparently contribute to chimpanzee density at Ngogo [30]. According to our study, sLFA species do not seem to play a role in Sebitoli chimpanzee density because they are absent from the chimpanzee diet. They may be difficult to identify at Sebitoli because they are scattered and not abundant, and therefore very difficult to census in the entire home range. However, particular skills developed by chimpanzees suggest they are able to categorize food resources based on specific functional classes (synchronous. asynchronous) [70]. Therefore, chimpanzees are able to gather information on diet availability and botanical features.

Five *Ficus* species at Sebitoli mostly fruit during high fruit abundance periods (that were initially classified as intermediate fruit abundance). Like tree diversity and *Ficus* species density, temporal availability of food resources (very short periods of relatively low or high fruit abundance, intermediate fruit availability, asynchronous species fruiting during time of low fruit availability) may also be a major factor explaining chimpanzee density at Sebitoli. Species providing fruits during periods of high fruit abundance (when fruit diversity is frequently high) at Sebitoli (N = 11), as at Ngogo (N = 9), seem to favor chimpanzee density. At some chimpanzee study sites, high quality foods are available across seasons [71], [72] and chimpanzees show only limited consumption of relatively low-quality fallback foods such as THV [73]. Chimpanzees can maintain high quality diets year round in cases of low seasonal variations (which is the case of Sebitoli) and high-quality fallback foods. Finally, in disturbed habitat such as regenerating forest that experienced logging activities in the past in Kibale, THV can reach high proportions and consequently offer a large diversity of terrestrial herbaceous vegetation as fallback food.

## Conclusion

In the site of Sebitoli, deeply impacted by human past and current activities, chimpanzees are dependent upon Fig. species, and we suggest that the high density of chimpanzees at the site is explained at least partially by temporal fruit availability as well as tree diversity. Owing to their fission-fusion social system, chimpanzees are capable of behaviorally coping with the restrictions on density imposed by the features of this relatively early stage regenerating forest (e.g., forage in small parties when using a small feeding patch in a site where biomass of feeding trees is low). The issue whether chimpanzees communities show a general capacity for resilience to anthropogenic influence and whether they take advantage of disturbed areas (THV, regenerating areas, crops) resulting from human activities inside and outside protected areas deserves further exploration in the future. The high density of chimpanzees at the Sebitoli site is surprising, given its geographical constraints, and past to present exploitation. However, our results suggest that chimpanzees may be able to circumvent the effects of these anthropogenic factors, and therefore their long-term impact may be relatively limited.

Between 1990 and 2000, forest cover decreased by 0.8% in Africa and Cental America, by 0.3% in Asia and Oceania and by 0.4% in South America while it increased in Europe (0.4%) and North America (0.1%) [74]. Primate populations and habitats are therefore highly subject to forest change. Empirical and quantitative information on their capacity for recovery, and the mechanisms through which they recover, is needed in a context of population growth and environment quality management to assess the system equilibrium. Integrating small scale analysis, inter-site comparisons and interdisciplinary methods in nature conservation plans could lead to a better understanding of possible co-existence between wildlife sustainability and human needs.

## Supporting Information

Figure S1 **Summary of methodology for classifying food ressources.**
(TIF)

Table S1 THV species consumed by Sebitoli chimpanzees.
(DOC)

Table S2 Direction compares BA sum and indicates p-value difference significativity (p-value ≤0.05; < or >) or non-significativity (≈) (SE: Standard Error).
(DOC)
