# Supplementary figures and images for: Suitable Habitats for Endangered Frugivorous Mammals: Small-Scale Comparison, Regeneration Forest and Chimpanzee Density in Kibale National Park, Uganda
Source: PLoS One. 2014 Jul 17;9(7):e102177. doi: 10.1371/journal.pone.0102177 (PMC4102508; doi:10.1371/journal.pone.0102177)

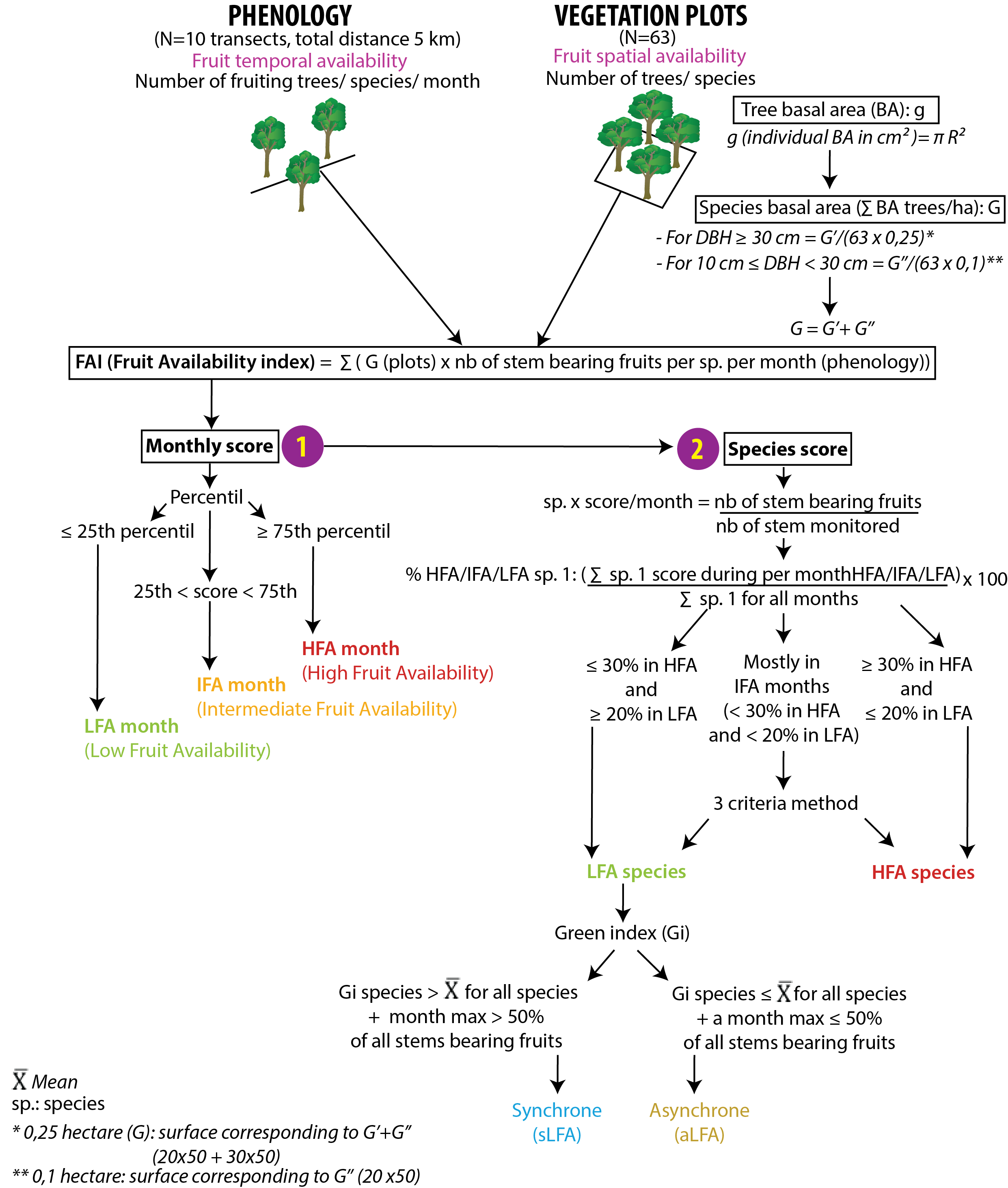

Supplement: Figure S1 — Summary of methodology for classifying food ressources. (TIF) [file pone.0102177.s001.tif]
