# Supplementary material for: Suitable Habitats for Endangered Frugivorous Mammals: Small-Scale Comparison, Regeneration Forest and Chimpanzee Density in Kibale National Park, Uganda
Source: PLoS One. 2014 Jul 17;9(7):e102177. doi: 10.1371/journal.pone.0102177 (PMC4102508; doi:10.1371/journal.pone.0102177)
Supplement: Table S1 — THV species consumed by Sebitoli chimpanzees. (DOC) [file pone.0102177.s002.doc]

Table S1. THV species consumed by Sebitoli chimpanzees.

|  | Pith | Stem | Flower | Leaf | Fruit | Total items |
| --- | --- | --- | --- | --- | --- | --- |
| *Acalypha ornata* |  |  |  | x |  | 1 |
| *Acanthus pubescens* | x | x |  |  |  | 2 |
| *Aframomum sp.* | x |  |  |  | x | 2 |
| *Centella alba* |  |  |  | x |  | 1 |
| *Cyphomoandra bataceae* | x |  |  |  | x | 2 |
| *Ensete ventricosum* | x |  |  |  |  | 1 |
| *Fern sp.* | x |  |  | x |  | 2 |
| *Hoslundia opposita* |  |  |  |  | x | 1 |
| *Jasminum sp.* |  |  | x | x |  | 2 |
| *Lepistemon owariensis* |  |  |  | x |  | 1 |
| *Pennisetum purpureum* | x |  |  |  |  | 1 |
| *Phytolacca dodecandra* |  |  |  |  | x | 1 |
| *Piper capense* | x |  |  |  |  | 1 |
| *Piper umbellatum* | x |  |  |  |  | 1 |
| *Solanesio manii* | x |  |  |  |  | 1 |
| *Triumfetta tomentosa* |  |  |  | x |  | 1 |
| *Urtica massaica* |  |  | x |  | x | 2 |
| Total items | 9 | 1 | 2 | 6 | 5 | 23 |
