# Supplementary material for: Suitable Habitats for Endangered Frugivorous Mammals: Small-Scale Comparison, Regeneration Forest and Chimpanzee Density in Kibale National Park, Uganda
Source: PLoS One. 2014 Jul 17;9(7):e102177. doi: 10.1371/journal.pone.0102177 (PMC4102508; doi:10.1371/journal.pone.0102177)
Supplement: Table S2 — Direction compares BA sum and indicates p-value difference significativity (p-value ≤0.05; < or >) or non-significativity (≈) (SE: Standard Error). (DOC) [file pone.0102177.s003.doc]

Table S2. Tree species most consumed by Sebitoli chimpanzees.

|  | Pith | Bark | Dead wood | Wax | Flower | Leaf | Sp. | Fruit | Total items |
| --- | --- | --- | --- | --- | --- | --- | --- | --- | --- |
| *Albizia grandibracteata* |  | x |  | x |  |  |  |  | 2 |
| *Aphania senegalensis* |  |  |  |  |  |  |  | x | 1 |
| *Beilschmiedia ugandensis* |  |  |  |  |  |  |  | x | 1 |
| *Cassipourea ruwensorensis* |  |  |  |  | x |  |  |  | 1 |
| *Celtis africana* |  | x |  |  |  | x |  |  | 2 |
| *Celtis gomphophylla* |  |  |  |  |  |  |  | x | 1 |
| *Celtis sp.* |  |  |  |  |  | x |  |  | 1 |
| *Chaetacme aristata* |  |  |  |  |  | x |  |  | 1 |
| *Chionanthus africanus* |  |  |  |  |  |  |  | x | 1 |
| *Chrysophyllum albidum* |  |  |  |  |  |  |  | x | 1 |
| *Chrysophyllum gorungosanum* |  |  |  |  |  |  |  | x | 1 |
| *Cordia africana* | x |  |  |  |  | x |  | x | 3 |
| *Cordia millenii* |  |  |  |  |  |  |  | x | 1 |
| *Croton sp.* |  |  |  |  | x |  |  |  | 1 |
| *Dasylepis eggelingi* |  |  |  |  |  |  |  | x | 1 |
| *Dombeya kirkii* | x |  |  |  |  |  |  |  | 1 |
| *Dovyalis macrocalyx* |  |  |  |  |  |  |  | x | 1 |
| *Drypetes gerrardii* |  |  |  |  |  |  |  | x | 1 |
| *Ehretia sp.* |  |  |  |  |  |  |  | x | 1 |
| *Euadenia eminens* |  |  |  |  |  |  |  | x | 1 |
| *Fagara sp.* |  |  |  |  |  |  |  | x | 1 |
| *Ficus asperifolia* |  |  |  |  |  | x |  |  | 1 |
| *Ficus brachypoda* |  |  |  |  |  |  |  | x | 1 |
| *Ficus conraui* |  |  |  |  |  |  |  | x | 1 |
| *Ficus cyathistipula* |  |  |  |  |  |  |  | x | 1 |
| *Ficus exasperata* |  |  |  |  |  | x |  | x | 2 |
| *Ficus mucuso* |  |  |  |  |  |  |  | x | 1 |
| *Ficus natalensis* |  |  |  |  |  |  |  | x | 1 |
| *Ficus ottoniifolia* |  |  |  |  |  | x |  | x | 2 |
| *Ficus sansibarica* |  | x |  |  |  |  |  | x | 2 |
| *Ficus saussureana* |  | x |  |  |  | x |  | x | 3 |
| *Ficus sur* |  |  |  |  |  |  |  | x | 1 |
| *Ficus thonningii* |  |  |  |  |  |  |  | x | 1 |
| *Ficus trichopoda* |  |  |  |  |  |  |  | x | 1 |
| *Ficus vallis-choudae* |  |  |  |  |  |  |  | x | 1 |
| *Millettia dura* |  |  |  |  |  |  | x |  | 1 |
| *Mimusops bagshawei* |  |  |  |  |  |  |  | x | 1 |
| *Monodora myristica* |  |  |  |  |  |  |  | x | 1 |
| *Myrianthus arboreus* |  |  |  |  |  |  |  | x | 1 |
| *Neoboutonia macrocalyx* |  |  | x |  |  |  |  |  | 1 |
| *Olea welwitschii* |  |  |  |  |  |  |  | x | 1 |
| *Parinari excelsa* |  |  |  |  |  |  |  | x | 1 |
| *Phoenix reclinata* | x |  |  |  |  |  |  | x | 1 |
| *Pouteria altissima* |  |  |  |  |  |  |  | x | 1 |
| *Prunus africana* |  |  |  |  |  | x |  | x | 2 |
| *Pseudospondias microcarpa* |  |  |  |  |  |  |  | x | 1 |
| *Tabernaemontana pachysiphon* |  |  |  |  |  |  |  | x | 1 |
| *Trema orientalis* |  |  |  |  |  |  |  | x | 1 |
| *Trichilia rubescens* |  |  |  |  | x | x |  |  | 2 |
| *Unknown sp. 001* |  |  |  |  | x |  |  |  | 1 |
| *Urera hypsiloides* | x |  |  |  | x | x |  | x | 4 |
| *Uvariopsis congensis* |  |  |  |  |  |  |  | x | 1 |
| Total items | 4 | 4 | 1 | 1 | 5 | 11 | 1 | 40 | 66 |
